# Diversity and transcription of proteases involved in the maturation of hydrogenases in *Nostoc punctiforme *ATCC 29133 and *Nostoc *sp. strain PCC 7120

**DOI:** 10.1186/1471-2180-9-53

**Published:** 2009-03-11

**Authors:** Ellenor Devine, Marie Holmqvist, Karin Stensjö, Peter Lindblad

**Affiliations:** 1 Department of Photochemistry and Molecular Science, The Ångström Laboratories, Uppsala University, Box 523, SE-751 20 Uppsala, Sweden

## Abstract

**Background:**

The last step in the maturation process of the large subunit of [NiFe]-hydrogenases is a proteolytic cleavage of the C-terminal by a hydrogenase specific protease. Contrary to other accessory proteins these hydrogenase proteases are believed to be specific whereby one type of hydrogenases specific protease only cleaves one type of hydrogenase. In cyanobacteria this is achieved by the gene product of either *hupW *or *hoxW*, specific for the uptake or the bidirectional hydrogenase respectively. The filamentous cyanobacteria *Nostoc punctiforme *ATCC 29133 and *Nostoc *sp strain PCC 7120 may contain a single uptake hydrogenase or both an uptake and a bidirectional hydrogenase respectively.

**Results:**

In order to examine these proteases in cyanobacteria, transcriptional analyses were performed of *hupW *in *Nostoc punctiforme *ATCC 29133 and *hupW *and *hoxW *in *Nostoc *sp. strain PCC 7120. These studies revealed numerous transcriptional start points together with putative binding sites for NtcA (*hupW*) and LexA (*hoxW*). In order to investigate the diversity and specificity among hydrogeanse specific proteases we constructed a phylogenetic tree which revealed several subgroups that showed a striking resemblance to the subgroups previously described for [NiFe]-hydrogenases. Additionally the proteases specificity was also addressed by amino acid sequence analysis and protein-protein docking experiments with 3D-models derived from bioinformatic studies. These studies revealed a so called "HOXBOX"; an amino acid sequence specific for protease of Hox-type which might be involved in docking with the large subunit of the hydrogenase.

**Conclusion:**

Our findings suggest that the hydrogenase specific proteases are under similar regulatory control as the hydrogenases they cleave. The result from the phylogenetic study also indicates that the hydrogenase and the protease have co-evolved since ancient time and suggests that at least one major horizontal gene transfer has occurred. This co-evolution could be the result of a close interaction between the protease and the large subunit of the [NiFe]-hydrogenases, a theory supported by protein-protein docking experiments performed with 3D-models. Finally we present data that may explain the specificity seen among hydrogenase specific proteases, the so called "HOXBOX"; an amino acid sequence specific for proteases of Hox-type. This opens the door for more detailed studies of the specificity found among hydrogenase specific proteases and the structural properties behind it.

## Background

Cyanobacteria evolved more then 2.0 billion years ago and were the first organisms to perform oxygenic photosynthesis [1,2]. They exist in many different shapes and forms e.g. unicellular, filamentous and colonial and can even form symbiosis with a variety of organisms [3]. Several cyanobacterial strains also have the ability to fix atmospheric nitrogen into ammonium, a process performed by the enzyme complex nitrogenase. Among filamentous cyanobacteria like *Nostoc *sp. strain PCC 7120 and *Nostoc punctiforme *ATCC 29133 (from now on referred to as *Nostoc *PCC 7120 and *Nostoc punctiforme*), both used in the present study, this process takes place in specialised cells called heterocysts in which a thick envelope and lack of photosystem II activity creates a nearly oxygen free environment for the nitrogenase [3,4]. The same nitrogenase is also a key player in the hydrogen (H_2_) metabolism by producing H_2 _as a by-product during the fixing of atmospheric nitrogen (N_2_). In addition, cyanobacteria may also possess distinct [NiFe]-hydrogenases.

The cyanobacterial hydrogenases can functionally be divided into two groups; uptake hydrogenases, dimeric HupSL, that consumes H_2_, and bi-directional hydrogenases, pentameric HoxYHEFU, that can both consume and produce H_2 _[3]. In the case of *Nostoc *PCC 7120 both hydrogenases may be present, while *Nostoc punctiforme *only contains the uptake hydrogenase [3,5].

The cyanobacterial uptake hydrogenase is closely connected to both the N_2_-fixing process and the occurrence of a nitrogenase, recycling the H_2 _and thereby regaining energy and electrons. The function of the bi-directional hydrogenase is more unclear and suggestions range from functioning as a mediator of reducing power during anaerobic conditions to it being part of respiratory complex I [3].

Both types of hydrogenases go through an extensive maturation process that involves several different accessory proteins. Even though much is still to be learned about this maturation process in cyanobacteria, comprehensive studies in other organisms like *Escherichia coli *have been performed [6,7]. Particularly the large subunit of [NiFe]-hydrogenase (HupL and HoxH in cyanobacteria) requires numerous accessory proteins responsible for metal transport, biosynthesis and insertion of the metal atoms nickel and iron into its active site. The genes encoding for these proteins are usually referred to as the *hyp*-genes and have been identified in many organisms including several cyanobacterial strains [3]. The Hyp-proteins are considered unspecific and there is usually only one set of *hyp*-genes irrespective of the number hydrogenases in a single strain [8,9]. It was recently suggested that a set of protein encoding genes within the extended *hyp*-operon of *Nostoc *PCC 7120 may be involved in the maturation of the small subunit of the cyanobacterial uptake hydrogenase [10].

The final step in the maturation process of the large subunit is a proteolytic cleavage of the C-terminal, which results in a conformational change, and the association of the large subunit to the small subunit [11,12]. The number of amino acids that are cleaved off varies between different hydrogenases and organisms but the cleavage always takes place after the conserved motif DPCXXCXXH/R resulting in the histidine being the new C-terminal amino acid [11-14]. Several experiments together with sequencing data have indicated that these putative proteases, contrary to the Hyp-proteins, are specific to different hydrogenases; not only to hydrogenases in different bacterial strains but also to different hydrogenases within the same strain [12,15]. In both *Nostoc punctiforme *and *Nostoc *PCC 7120 putative proteases have been identified through secondary and tertiary structure alignments [16]. The protein product of the gene *hupW *is believed to process HupL (the large subunit of the uptake hydrogenase) and can be found in both cyanobacterial strains. *Nostoc *PCC 7120 however, which in addition harbours a bi-directional hydrogenase, also contains *hoxW *whose protein product is believed to be involved in the processing of HoxH [5,16].

It is still unknown exactly how the recognition of the different hydrogenases takes place and which part(s) of the protease determines specificity. A crystal structure of a large subunit- protease complex is still not yet available from any organism. However, the protease HupD from *E. coli *has been crystallised giving vital clues about its function [17]. The importance of Ni-incorporation into the active site for any cleavage to occur has been addressed [13,18,19] and together with amino acid replacement experiments, it has been shown that nickel is an important substrate recognition motif. In addition the protease binds directly to the metal [17,19] and the crystal structure of HybD in *E. coli *showed that three amino acids; Glu16, Asp62 and His93, are most likely to be involved in the metal binding [17].

Contrary to the lack of functional studies of cyanobacterial hydrogenases extensive studies have been done on the transcriptional regulation of cyanobacterial hydrogenases and their accessory genes [3]. Several putative binding sites of different transcription factors have been reported in connection with the uptake hydrogenase such as FNR (fumarate-nitrate reduction) in *Anabaena variabilis *and the global nitrogen regulatory protein NtcA in *Nostoc punctiforme, Lyngbya majuscule *CCAP 1446/4 and *Gloeothece *sp. strain ATCC 27152 and IHF (integrated host factor) in *Nostoc punctiforme *and *Lyngbya majuscule *CCAP 1446/4 [3]. Participation by the transcription factor NtcA fits in well with the known connection between the uptake hydrogenase and N_2 _fixation. Further it has been shown that the uptake hydrogenase is only transcribed under N_2_-fixing conditions and in connection with heterocyst formation [20,21].

The genes encoding the bi-directional hydrogenase, contrary to the uptake hydrogenase, are transcribed in both heterocysts and vegetative cells and under both non N_2_- and N_2_-fixing conditions [3]. So far, two transcription factors have been identified in connection with the bi-directional hydrogenase, LexA and an AbrB-like protein [22-24].

In the present study we investigate the transcriptional regulation of the genes encoding hydrogenase specific proteases *hupW *in *Nostoc punctiforme *and *hupW *and *hoxW *in *Nostoc *PCC 7120, under both N_2_-fixing and non N_2_-fixing conditions. In addition, we address the question of the diversity, specificity and evolution of the hydrogenase specific proteases in cyanobacteria.

## Results

### Diversity of cyanobacterial hydrogenase specific proteases

**Figure 1:**
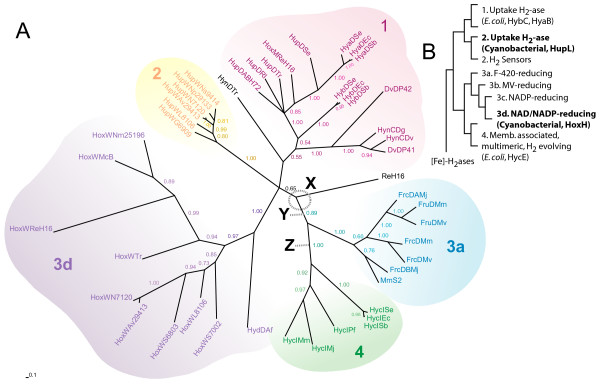
**Unrooted phylogenetic tree of hydrogenase specific proteases**. The phylogenetic tree of hydrogenase specific proteases from the MrBayes analysis including the different subgroups they may be divided into. The proposed subgroups for each protease are marked in the figure; 1 (red), 2 (orange), 3a (blue), 3d (purple), 4 (green) and unknown (black). X: The point in the phylogenetic tree when horizontal gene transfer occurred. Y/Z: Suggested positions of root. **B**. The phylogenetic tree of hydrogenases adapted from Vignais et al 2004 [25]. Type 2a (HupL) and 3d (HoxH) hydrogenases which can be found in cyanobacteria are marked in bold. The phylogenetic tree was obtained using MrBayes analyses and the claude credibility values are given beside each branch. For abbreviations see Table 2.

To examine the diversity of hydrogenase specific proteases and their relationship to each other, in cyanobacteria and other microorganisms, a phylogenetic tree was constructed using both PAUP and MrBayes analysis. Since no suitable outgroup has been found for the proteases at this stage, a non-rooted tree was constructed including claude creditability values. The resulting tree from the MrBayes analysis revealed several subgroups among the hydrogenase specific proteases, which correlates with respective hydrogenase group according to Vignais et al [25] (Figure 1);

1. Bacterial proteases (cleaves group 1 hydrogenases)

2. Cyanobacterial proteases, HupW type (cleaves group 2 hydrogenases)

3. Bacterial and Archaean proteases

a. Archean proteases (cleaves group 3a hydrogenases)

d. Bacterial proteases, HoxW type (cleaves group 3d hydrogenases)

4. Bacterial and Archaean proteases, Hyc type (cleaves group 4 hydrogenases)

The phylogenetic groups of the hydrogenase specific protease have been named according to the nomenclature used for [NiFe]-hydrogenase.

The result from the PAUP analysis is less resolved but supports the result from MrBayers analysis with some minor differences within group 3d (HoxW in *Synechocysis *sp. strain PCC 6803 and HoxW in *Synechococcus *sp. strain PCC 7002 are shown as more closely related).

An extended phylogenetic tree was also constructed containing more strains including hydrogenase specific proteases cleaving type 3b-hydrogenases. This tree was unfortunately less reliable and far from robust with several weak nodes (Additional file 1 and Additional file 2). However the result showed putative group 1 proteases and putative group 3b proteases as less clustered and instead spread around point X (Figure 1 and Additional file 1).

### Transcriptional studies of *hupW *in *Nostoc punctiforme *ATCC 29133 and *Nostoc *sp strain PCC 7120

**Figure 2:**
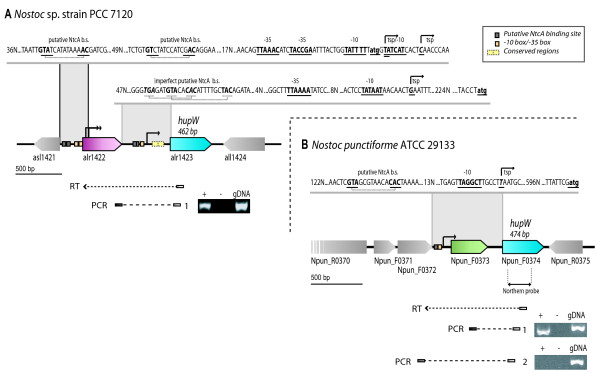
**Northern blot analysis of *hupW***. Northern blot analysis of the relative amount of *hupW *transcripts of *Nostoc *PCC 7120 and *Nostoc punctiforme *under different growth conditions, using a probe against *hupW *in *Nostoc punctiforme*. The positions of rRNAs are indicated, as seen on gel. The equal loading of the RNA were analyzed by determine the relative amount of *rnpB *transcripts.

**Figure 3:**
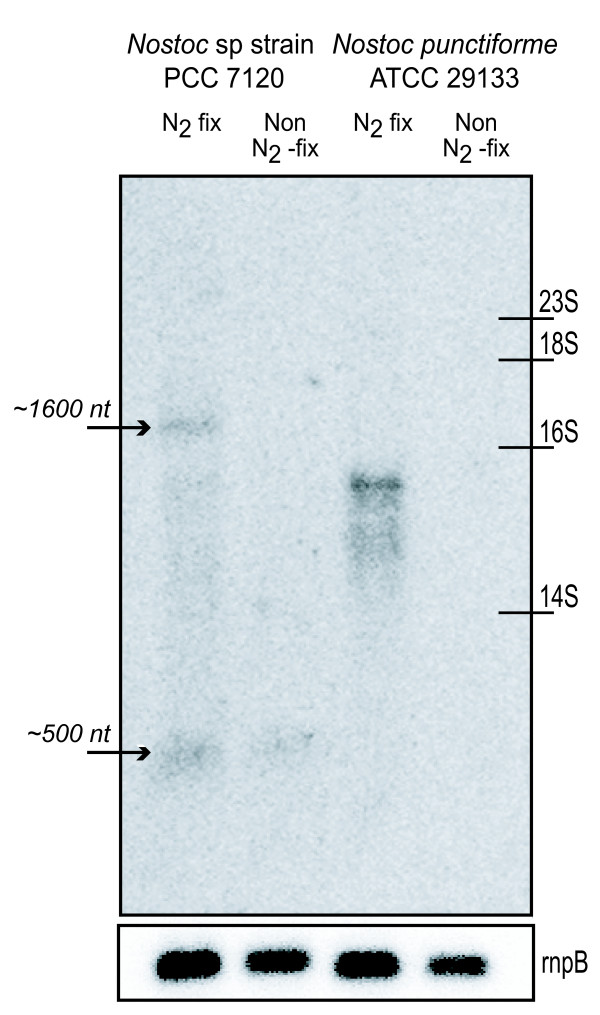
**Illustrations of the *hupW *operons**. The *hupW *operon and surrounding genes in *Nostoc *PCC 7120 and *Nostoc punctiforme*. **A**. The transcription start point (TSP) and promoter region of *hupW *in *Nostoc *PCC 7120 together with the result from the reverse transcription (RT) reaction and subsequent PCRs. The positions of primers used in the experiments are shown (Table 1). (+): PCR-fragment, (-): negative control without RT enzyme, gDNA: positive control with gDNA. **B**. Schematic presentation showing TSP and promoter region of *hupW *together with RT-PCR detection of *hupW *transcripts in *Nostoc punctiforme*. The positions of primers used are shown (Table 1). (+): PCR-fragment, (-): negative control without RT, gDNA: positive control with gDNA. Results of PCR were visualized on a 1% agarose gel.

Northern hybridisations were performed of *hupW *in both *Nostoc punctiforme *and *Nostoc *PCC 7120 using both N_2_-fixing and non N_2_-fixing cultures (Figure 2). The results from *Nostoc *PCC 7120 revealed two transcripts. The first is shorter (approx. 500 nt) and present under both N_2_-fixing and non N_2_-fixing conditions, while the second longer transcript (approx. 1600 nt) is only present under N_2_-fixing conditions. The size of the longer transcript is comparable with the size of a two-gene operon containing *hup*W together with the upstream gene alr1422, a gene of unknown function (Figure 3a). RT-PCR confirmed that the two genes are co transcribed (Figure 3a). Additional 5'RACE experiments revealed three TSPs whereby the first is located 234 bp upstream of *hupW*. Succeeding bio-informatic studies identified a putative σ^70^-like -10 and -35 box (Figure 3a) (TATAAT respectively TTAAAA) and two imperfect putative NtcA binding sites (TGAN_8_CAC and GTAN_12_TAC). By running the complete intergenic region in BLAST at Cyanobase two conserved regions were also discovered. Both can be found in the intergenic regions of several genes in *Nostoc *PCC 7120 and *Anabaena variabilis *ATCC 29413 (data now shown). Their function is unclear but one of them shows similarity to the consensus sequence WATCAANNNNTTR from the previously described IHF binding sites [26]. The second and third TSPs were identified inside the gene alr1422, 4 bp and 14 bp downstream of the putative translation start site. A new putative translation start site within the same frame was found 115 bp downstream from the previously suggested start site. By analysing the sequence of the promoter region a -10 box (TATTTT and TATCAT), a -35 box (TTAAAC and TACCGA) and two putative NtcA binding sites (GTAN_8_AAC/GTN_10_AC) 147/157 bp and 62/72 bp upstream of the two TSPs were also identified.

For *Nostoc punctiforme *a transcript of *hupW *of about 1300 nt, is only present in N_2_-fixing cultures (Figure 2). 5'RACEs identified a single TSP 607 bp upstream of *hupW *in *Nostoc punctiforme*, together with a σ^70^-like -10 box sequence (TAGGCT) and a putative NtcA binding site (GTAN_8_CAC) located 40 bp upstream from the TSP (Figure 3b). The resulting transcript includes the upstream gene Npun_F0373, which was confirmed by RT-PCR using primers for the subsequent PCR covering the intergenic region and agrees with the result from the Northern blot experiments (Figure 2 and 3b).

### *In silico *analysis of alr122 and Npun_F0373 in *Nostoc *sp. strain PCC 7120 and *Nostoc punctiforme *ATCC 29133

Homologues to alr1422 in *Nostoc *PCC 7120 are present in two other strains, *Anabaena variabilis *ATCC 29413 (ava3972) and *Trichodesmium erythraeum *IMS101 (tery_3492). It shows no transmembrane regions or domains that would give an indication of its function.

**Figure 4:**
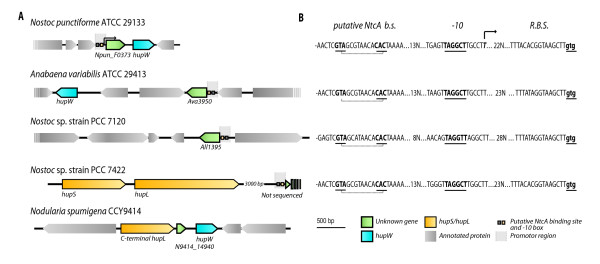
**Npun_F0373 and homologues**. Schematic picture showing Npun_F0373 in *Nostoc punctiforme *and its homologues in other strains (*Anabaena variabillis *ATCC 29413, *Nostoc *PCC 7120, *Nostoc *sp. strain PCC 7422, *Nodularia spumigena *CCY 9414), all indicated as "unknown gene". The promoter region of all strains (detailed in B) is highlighted in gray. **B**. The putative promoter regions of NpunF0373 and its homologues in other cyanobacterial strains show preserved putative NtcA binding sites, -10 box, TSP and ribosomal binding sites (RBS). The only strain lacking the promoter region is N9414_14940 of *Nodularia spumigena *CCY 9414, probably due to co-transcription with the C-terminal of *hupL*.

The gene Npun_F0373 is of unknown function but a search with NCBI BLAST revealed four homologues in other microorganisms, all cyanobacterial; *Nostoc *PCC 7120, *Anabaena variabilis *ATCC 29413, *Nodularia spumigena *CCY 9414 and in *Nostoc *sp. PCC 7422 (Figure 4, Additional file 3). In *Nostoc *sp. strain PCC 7422 only parts of the genome are sequenced and in the 5'end of GenBank accession number AB237640 the first 63 bp of the gene can be identified. The gene is truncated in *Nodularia spumigena *CCY 9414 but is intact in the other strains and in two cases (*Nostoc punctiforme *and *Nodularia spumigena *CCY 9414) it is located directly upstream of *hupW *and/or the uptake hydrogenase genes. Alignments of the promoter sequence of these genes show highly conserved promoter regions, all containing putative NtcA binding sites, -10 box, putative Shine-Dalgarno sequence and even suggests a putative TSP for four out of the five genes (the gene Npun_F0373 homologue in *Nodularia spumigena *CCY9414 is probably transcribed with the upstream gene, *hupL*) (Figure 4). Bio-informatic studies of Npun_F0373 propose a transmembrane region between amino acids 84–105 but showed no other domains or sites giving clues to its function. However, when comparing strains that either harbour or lack the gene, it was found that among the strains containing Npun_F0373 and its homologues, the ability to form heterocysts is a shared feature (Additional file 4).

### Transciptional studies of *hoxW *in *Nostoc *sp strain PCC 7120

**Figure 5:**
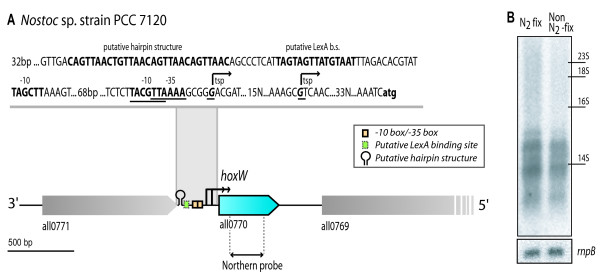
**The transcript of *hoxW *in *Nostoc *PCC 7120**. **A**. Schematic presentation of *hoxW *and surrounding genes in *Nostoc *sp. strain PCC together with nucleotide sequence of putative promoter region for *hoxW*. **B**. Northern blot analysis of the relative amount of *hoxW *transcripts of *Nostoc *PCC 7120 under different growth conditions. The positions of rRNAs are as seen on the gel. The experiment were done in two biological replicate and the equal loading of the RNA was analyzed by determine the relative amount of *rnpB *transcripts.

*hoxW *is located between the genes all0771 (4-hydroxyphenylpyruvate dioxygenase) and all0769 (acetyl-CoA synthetase), both with no known relationship to H_2 _metabolism, and around 4.7 kbp downstream of the *hoxHYU *operon [23] on the opposite strand (Figure 5).

Northern blot hybridisation of *hoxW *was performed using RNA isolated from both N_2_-fixing and non N_2_-fixing cultures indicating an increased level of *hoxW *under N_2_-fixing conditions and revealing several transcripts ranging from ~1000-500 nt (Figure 5b). This was confirmed by 5'RACE experiments that showed TSPs at both 44 bp and 70 bp upstream of *hoxW*. When analysing the promoter region, a σ^70^-like -10 box (TAGCTT) was identified for the TSP, 70 bp upstreams of *hoxW*, but no -35 box while the TSP, 44 bp upstream of *hoxW*, contains a putative -35 box (TTAAAA) but no clear -10 box (Figure 5a).

When analysing the complete intergenic region between *hoxW *and its upstream gene all0771 two conserved regions appeared (Figure 5a). Both regions can be found in between genes in numerous cases especially in the genome of *Nostoc *PCC 7120 and *Anabaena variabilis *ATCC 29413. The first conserved region, situated 204–231 bp upstream of *hoxW*, consists of four repeats, which when run through Mfold forms a putative hairpin (dG = -10.21). The second region is located 162–195 bp upstream of *hoxW *and its sequence TAGTAGTTATGTAAT(N_12_)TAGCTT shows resemblance to a LexA binding site, according to the previously defined motif RGTACNNNDGTWCB together with a putative -10 box [27].

### Specificity of HupW and HoxW in cyanobacteria

**Figure 6:**
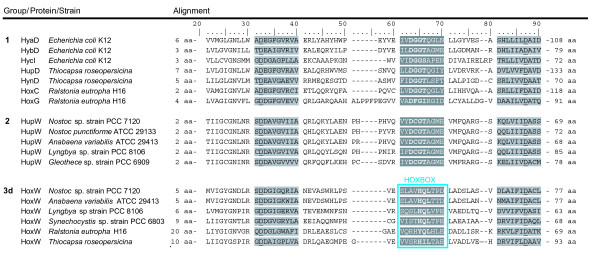
**Alignment of hydrogenase specific proteases from group 1, 2 and 3d in the phylogenetic tree (Figure 1)**. Two conserved asparagines (underlined) are believed to be involved in binding to the nickel of the large hydrogenase subunit. Between these asparagines there is a conserved area of unknown function, the so called "HOXBOX". As seen in this figure, although differing among organism, it is in fact conserved within groups of hydrogenase specific proteases i.e. proteases of 3d/HoxW-type. Conserved asparagine (D) containing-regions; light grey, conserved region of unknown function (D(G/C)GT); dark grey and conserved region of unknown function (H(Q/I)L); dark grey, underlined.

**Figure 7:**
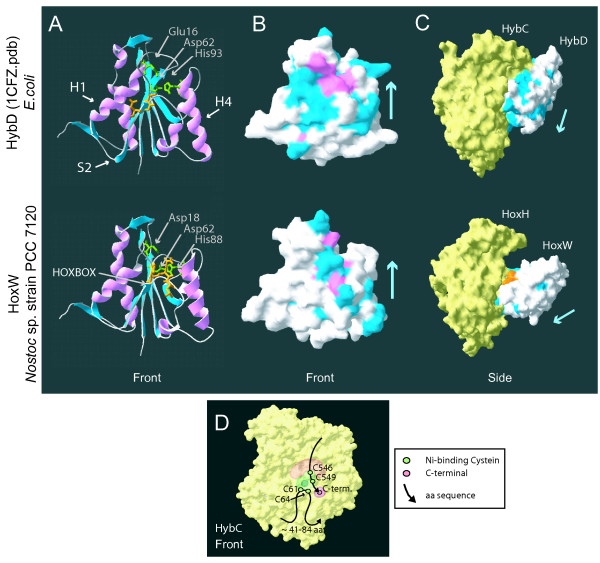
**HybD (**1CFZ**.pdb) from *E. coli *and the 3D-structure model of HoxW from *Nostoc *PCC 7120**. Illustration showing the crystallised structure of HybD (1CFZ.pdb) from *E. coli *(top) and the 3D structure model of HoxW from *Nostoc *PCC 7120 (bottom). **A**. Ribbon diagram of HybD (*E.coli*) and HoxW (*Nostoc *PCC 7120). Colour guide; green: amino acids believed to be involved in binding to the nickel in the active site of the large subunit, orange: the differently conserved residues i.e. the "HOXBOX" in HybD (DGG) and HoxW (HQL). Abbreviations; H: α-helix, S: β-sheet. **B**. The position of conserved amino acid residues on the surface of a representative of hydrogenase specific proteases from group 1 (HybD-1CFZ.pdb) and 3d (HoxW-3D model). Colour guide; red: residues conserved among all (100%) of the strains within a group, blue: residues found to be conserved or similar among 80% of the strains in each group. **C**. Protein-protein docking result of hydrogenase specific proteases to the large subunit of the [NiFe]-hydrogenase. HybC (large subunit) and HybD (protease) from *E. coli*. HoxH (large subunit) and HoxW (protease) from *Nostoc *PCC 7120. Colour guide; orange: conserved residues, i.e. the "HOXBOX" region, blue: identical and similar residues shared by 80% of the strains in group 1 and group 3d respectively. Light blue arrow indicates direction as seen in (B). Three of the structures (HybC, HoxH and HoxW) were modelled by using the online program SWISS-MODEL. **D**. Space filling structure of HybC (*E. coli*). Colour guide; green: active site with the four cysteins involved in the binding of nickel and iron, red: the C-terminal histidine (His552), orange: region on the large subunit which might be in contact with the HOXBOX.

To address the protease specificity an alignment of protein sequences was performed to search for conserved regions specific to each protease group, HupW and HoxW (group 2 and 3d, Figure 1), in cyanobacteria. This study revealed that one of the conserved regions among the proteases is highly dissimilar when comparing HupW and HoxW in cyanobacteria (Figure 6 and Figure 7a). In most proteases, including HupW, this region consists of the sequence D(G/C/F)GT (aa 41–44 in HupW of *Nosotoc *PCC 7120) while among the HoxW proteases it is replaced by the sequence H(Q/I)L (aa 42–44 in HoxW of *Nostoc *PCC 7120) (the latter now on referred to as the HOXBOX).

To get a better understanding of this region and its possible function bio-informatic work was performed targeting conserved and similar amino acids on the surface of putative HoxW and putative HupW in *Nostoc *PCC 7120 and HybD in *E. coli *together with protein-protein docking experiments using the docking algorithm BiGGER. The studies showed that the conserved residues are not evenly distributed but clustered around the proposed nickel binding residues Glu16 and His93 (HybD – *E. coli*) [17] and around the conserved "HOXBOX" region for all three cases. In HupW and HybD conserved surface areas could also be found along alpha helix 1, beta sheet 2 and alpha helix 4 [16,17] (Figure 7a–b).

Protein docking experiments resulted in 11 hits for HybC-HybD (*E. coli*), 84 hits for HybB-HynC (*Desulfovibrio vulgaris str. Miyazaki F*) and 28 hits for HoxH-HoxW (*Nostoc *PCC 7120). The best hit for HybD in *E. coli *and HoxW in *Nostoc *PCC 7120 can be seen in Figure 7c, a target-probe complex whereby the HOXBOX of the protease is in a less favourable position for C-terminal cleavage. This means that the HOXBOX is either facing away from the C-terminal or that other residues are blocking making it difficult for physical contact to occur without major conformation changes. This was the case for 70% of the hits and the average distance of Gly42/His42 (HybD/HoxW) in the HOXBOX to the last amino acid of the C-terminal was around 17–20 Å. The majority of the hits indicated that the HOXBOX region and the areas around alpha helix 1, beta sheet 2 and alpha helix 4 are in close interaction with the large subunit of the hydrogenase. This is especially true for the HybC-HybD complex while HoxH-HoxW showed a preference for a more narrow interaction with only the closest residues around Asp16 and His88 and the HOXBOX involved in the contact with HupL. The preferred docking result for HybD in *E. coli *and HoxW in *Nostoc *PCC 7120 reflects the results from the studies of the conserved residues as can be seen when comparing Figure 7b and Figure 7c.

## Discussion

### Diversity of cyanobacterial hydrogenase specific proteases

Previous phylogenetic studies of hydrogenases in different microorganisms [3,28,29] clearly divide the proteins into four classes [28,29]. One of the most extensive studies, using over 80 microorganisms, showed that the large and the small subunit of the hydrogenase enzyme evolved together and have been two tightly connected subunits for probably all of their evolutionary history [25]. When comparing the evolution of hydrogenases with the present study of hydrogenase specific proteases some striking resemblances appear which indicate a similar development and co-evolution between the large subunit of the hydrogenases and their specific proteases (Figure 1).

Within the phylogenetic tree of the hydrogenase specific proteases similar groups appear as seen among the hydrogenase subunits. This is especially true for the proteases in group 1, 2, 3a and 4. Just as the hydrogenase subunit HycE in *E. coli *(group 4) is most closely related to the archean hydrogenases (group 3) so is its hydrogenase specific protease HycI (group 4) most closely related to group 3 proteases. The resemblance between the phylogenetic trees suggests that the co-evolution between the hydrogenase and the hydrogenase specific protease is of ancient origin and an explanation for this might be found in the mechanism of the cleavage process. It has previously been suggested that a conformational recognition takes place between the protease and the large subunit [19] which may through the years enhanced the specificity seem among proteases.

The Hox-specific proteases of group 3d are the exception and can be found as an independent group (Figure 1). Further studies, even though not as robust, also show proteases of 3b type and Additional proteases of group 1 type being spread either individually or on branches around point X (Additional file 1). These results contradict previous evolutionary studies of their respective hydrogenases which have placed group 3b/3d hydrogenases as clearly defined subgroups within group 3 [NiFe]-hydrogenases [29]. By comparing the [NiFe]-hydrogenase phylogenetic tree with the protease phylogenetic tree presented in this study, it also becomes apparent that neither group 1, 2 or 3d would be the deepest branch in a rooted version of the tree. Such a tree would suggest that proteases within the groups 3b/3d developed before the proteases of group 3a and 4, which seems far-fetched since proteases of group 3a and 4 type cleaves hydrogenases that are deeper branched then the 3b/3d hydrogenases.

We therefore suggest that the placement of HOX-specific proteases (3d) and the scattered result of 3b proteases in the phylogenetic tree may be the result of horizontal gene transfer (HGT). HGT is today seen as a major force in evolution and has occurred numerous times between archaea and bacteria [30-33]. Within prokaryotes almost no gene family is untouched by HGT [34] and there are also numerous cases of HGT within cyanobacteria [35]. [NiFe]-hydrogenases have not been spared from this mechanism and an archaeal organism is believed to be the origin of the *Ech*- hydrogenase in *Thermotoga maritima *[36].

**Figure 8:**
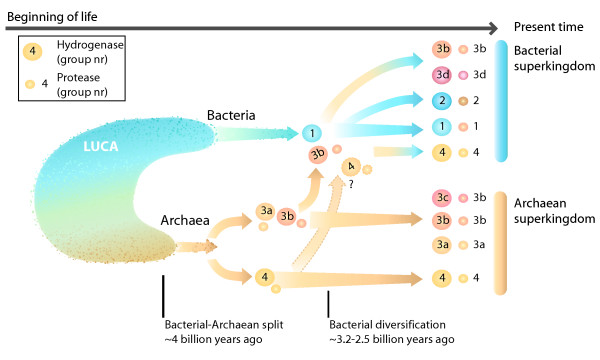
**Illustration showing the proposed horizontal gene transfer (HGT) of a type 3 hydrogenase/protease from an archaeal organism to a bacterial organism**. The result form the phylogenetic tree indicates that it has been at least one major HGT event within the evolution of [NiFe]-hydrogenases and the hydrogenase specific proteases. Our results suggest that the root may be placed between group 3a and 4 of the hydrogenase specific proteases which would mean that the proteolytic cleavage of the hydrogenase large subunit by a protease originated within the archaean superkingdom. This illustration indicates the proposed HGT that transferred the protease to bacteria, which could then have been incorporated to the maturations process of type 1 and 2 hydrogenases. This theory does not rule out that additional HGT might have occurred and in this illustration type 4 hydrogenases within proteobacteria, together with their specific protease, are shown as the result of a similar HGT. This is still unclear though and the type 4 hydrogenases might have existed in both bacteria and archaea from the start. Large circle; hydrogenase, small circle; protease, red/orange colour; suggested archaean origin, blue colour; suggested bacterial origin.

By comparing the phylogenetic tree of hydrogenases and their specific protease and assuming that the [NiFe]-hydrogenase and its specific protease have evolved together the most likely scenario is that an early group 3 [NiFe]-hydrogenase with or without its specific protease was transferred, most probably from an archaeal organism to a bacterial. If we assume that the type 3 hydrogenase and the protease transferred together then this indicates that most likely the root of the tree should be placed between group 3a and 4 (point Z; Figure 1) and that the protease transferred is the ancestor of all type 1, 2 and 3d proteases (Figure 8). If we assume the opposite, (that the hydrogenase transferred alone), then the root should instead be placed between type 1/2/3d and type 3a/4 proteases (point Y; Figure 1) and the transferred hydrogenase must have incorporated an already existing type 1 protease to its maturation process. The scattered impression of type 1 and 3b proteases from the less robust phylogenetic tree with additional hydrogenase specific proteases (Additional file 1) could be the result e.g. older phylum branching off close to the HGT point, poor resolution of the phylogenetic tree or by additional HGT and so does not contradict our proposed theory of HGT. Rooting the tree with an outgroup; germination protease (GPR), the closest relative to the [NiFe]-hydrogenase specific proteases, (data not shown) placed the root between group 3a and 4 suggest that the first scenario, a root between group 3a and 4, is more plausible (point Z; Figure 1). However, all attempts at rooting the tree resulted in very unstable phylogenetic trees. When considering both GPR endopeptidase function (bacterial spoluration) and taxonomic location (bacterial phylum of firmicutes only) it is plausible that the [NiFe]-hydrogenase specific proteases are instead the ancestor of GPR, making any tree with GPR as outgroup unreliable.

Based on the tree of life we also propose that the HGT of probably a 3b similar type protease/hydrogenase most likely took place before the diversification of the bacterial phylum and group 1 hydrogenases. [37,38]. By comparing our result with genomic timescales of prokaryotic evolution we can even suggest a time for the event of around 3–3.5 billion years ago [39,40]. This is based on that the archaeal phylum and classes started to evolve earlier (between 4-3 billion years ago) then the bacterial (~3-2.5 billion years ago) and the proposition that methanogenesis was one of the first metabolical pathways to be developed [39]. Since group 3a-3b hydrogenases, have previously been shown to be connected to methanogenesis [29] this data supports our suggestion of an early differentiation of group 3 hydrogenases. It should be noted that this proposed theory does not contradict previous suggestions of an early pre-LUCA existence and diversification of hydrogenases but rather clarifies the picture [29,41]. The effect this proposed HGT had on bacterial evolution is not clear but HGT in general may have had a significant effect on the diversification of bacterial species by introducing new metabolic pathways and traits [42,43].

Large-scale molecular genetic analysis of the DNA sequence (like studies of gene order and G-C content) could give a clearer picture however, because the HGT might have occurred more then 3 billion years ago mechanisms like amelioration will most likely have erased all evidence.

### Transcriptional studies of *hupW *in *Nostoc punctiforme *ATCC 29133 and *Nostoc *sp strain PCC 7120

It is interesting that *hupW *in both *Nostoc punctiforme *and *Nostoc *sp. strain PCC 7120 are only or mainly transcribed under N_2_-fixing conditions even though it is not a surprising discovery. The same pattern has been observed for the uptake hydrogenase whose function has previously been connected to N_2_-fixing [3]. This suggests that the *hupW *proteases are under the same or similar transcriptional regulation as the hydrogenases they cleave. This expression pattern could be explained by the putative NtcA binding sites in the promoter region of *hupW *in both *Nostoc punctiforme *and *Nostoc *PCC 7120 (Figure 3b). NtcA binding sites have been found upstream of *hupSL *in *Gloeothece *sp. ATCC 27152 [44], *Nostoc punctiforme *[45], *Lyngbya majuscule *CCAP 1446/4 [46] and *Anabaena variabilis *ATCC 29413 [47], and putative binding sites have been observed upstream of the *hyp*-genes in *Nostoc punctiforme *[48].

The two putative NtcA binding sites (TGAN_8_CAC and GTAN_12_TAC) identified upstream of the TSP of *hupW *in *Nostoc *PCC 7120 are imperfect when compared with the sequence signature of NtcA (GTAN_8_TAC) [49,50]. These sites are therefore likely to have none or a very weak binding affinity to NtcA and the two conserved regions observed downstream of the TSP may be the target of additional transcription factors. Sequences similar to these conserved regions were also found in the intergenic regions of several other genes in *Nostoc *PCC 7120 and *Anabaena variabilis *ATCC 29413 (data not shown) and one of the conserved regions shows resemblance to an IHF binding site and the consensus sequence WATCAANNNNTTR [26,51]. Binding sites for IHF have previously been found in the promoter region of *hupSL *in *Nostoc punctiforme *[45] and *Lyngbya majuscula *[46] but have also been observed upstream of the *hup *genes in *Bradyrhizobium japonicum *[52], the *nif *genes in purple bacteria [53] and the *nif *operon in *Anabaena azollae *[54].

### Transciptional studies of *hoxW *in *Nostoc *sp strain PCC 7120

Contrary to the *hupW *regulation, the result from the Northern blot studies of transcript level on *hoxW *in *Nostoc *PCC 7120 showed only a minor difference between non N_2_-fixing (lower) and N_2_-fixing conditions (higher). Considering the very small difference seen in transcript level the main function of the bi-directional hydrogenase and its specific protease indicate that it is not connected to N_2_-fixation. Studies of the transcript levels of the bi-directional hydrogenase subunit *hoxH*, when shifted from non N_2_-fixing to N_2_-fixing (*Nostoc muscorum*) or to N_2 _limiting (*Gloeocapsa alpicola*) conditions, shows either no effect (*Nostoc; *[20]) or very small effect (*Gloeocapsa; *[55]). However further studies of the bi-directional hydrogenase activity in *Gloeocapsa alpicola *actually showed significantly increased activity even though the relative abundance of *hoxH *(and *hoxY*) transcript did not change [55].

Conserved regions were identified in the promoter region of *hoxW*. The first region, containing a short tandemly repeated repetitive (STRR) sequence, has the ability to form a hairpin loop which is not unusual in filamentous cyanobacteria and has been found between *hupS *and *hupL *in *Anabaena variabilis *ATCC 29413, *Nostoc *PCC 7120, *Nostoc punctiforme *and *Lyngbya majuscula *CCAP 1446/4 [46,56,57]. In cyanobacteria they are usually made up of 7 bp repeats and even if their function is still not known they may be involved in increasing transcript stability or confer a translation coupling between genes [3,56,58]. Hairpin structures in the DNA sequence can also result in pauses during transcription or even act as a termination site [26]. The latter is a more likely scenario in this case since the putative hairpin is positioned close to the 3' end of the previous gene all0769 (4-hydroxyphenylpyruvate dioxygenase), which is not co-transcribed with *hoxW*.

The second conserved region in the *hoxW *promoter region shows a strong resemblance to the consensus sequence RGTACNNNDGTWCB of a LexA binding site [27]. LexA has previously been shown to bind to the promoter region of the *hox*-genes in *Synechocystis *sp. strain PCC 6803 [22,59] and *Nostoc *PCC 7120 [23], and the *hyp*-genes in *Lyngbya majuscula *CCAP 1446/4 [60].

### Specificity of HupW and HoxW in cyanobacteria

An alignment of the deduced amino acid sequence of several groups of proteases revealed that one of the conserved regions found in hydrogenase specific proteases was replaced by a new, unique region in HoxW proteases (group 3d), the so called HOXBOX (aa 42–44 in HoxW, *Nostoc *PCC 7120). This novel observation of a conserved group specific region may be an important finding for the understanding of the specificity and function of hydrogenase specific proteases. The function of this region in hydrogenase specific proteases has previously been under speculation with some suggesting that it functions as a catalytic site for the proteolytic cleavage [17,61] and others that it is involved in substrate binding [17]. Amino acid replacement, whereby Asp38 in HycI in *E. coli *was changed to an asparagine showed no effect on the cleavage process [62] which of course does not rule out that other parts of this region might be of importance.

In silico location studies of conserved surface residues of different proteases identified that the conserved amino acids are unevenly distributed on the surface and concentrated to certain regions (Figure 7b). To find conserved residues around the proposed nickel binding amino acids Glu16 and His93 (HybD – *E. coli*) is to be expected considering the importance of these residues for substrate binding. Interestingly, conserved residues were also observed around the HOXBOX region and further on along alpha helix 1, beta sheet 2 and alpha helix 4 [16,17], especially in group 1 and 2 of the proteases. This could be due to their importance for the overall structure of the protein but could also indicate that these areas are involved in either cleavage function or docking between the protease and the large hydrogenase subunit. The latter theory coincides well with the result from the protein docking studies (Figure 7c). The same areas that contain a high degree of conserved residues were in the docking result often seen in close contact with the hydrogenase. The protein docking results, performed with hydrogenases and proteases from several organisms, places the HOXBOX alternatively the corresponding region continuously in unfavourable positions for C-terminal cleavage making its possible function as a catalytic site unlikely. Added to the already mentioned observation that this region exist in two variations (i.e. the HOXBOX or D(G/C/F)GT) it seems more reasonable it is involved in substrate binding and recognition and might even be important for the proteases specificity.

It should be mentioned that these protein-docking studies are mostly performed with 3D-models constructed through protein threading since no crystallised hydrogenase and protease exist from the same organism. Even though the proteins used in this study are related, the sequence identities are sometimes low (20–25%) but increases in the putative docking areas (30–40%). The large subunit of the hydrogenase is also believed to exist in an open conformation, which probably makes the nickel associated to the active site of the hydrogenase accessible for the protease [7]. An open conformation could have an immense effect on any kind of protease-hydrogenase interaction but is with today's knowledge impossible to predict.

## Conclusion

An understanding of the transcriptional regulation of hydrogenase specific proteases in cyanobacteria is starting to emerge. It suggests that the hydrogenase specific proteases in cyanobacteria are under very similar regulatory control as the hydrogenases they cleave. The two proteins also appear to have a close physical interaction during the cleavage moment, which could explain the specificity seen among proteases and the resemblance seen between the protease and the hydrogenase phylogenetic trees, and this interaction might be of very ancient origin. After comparing the phylogenetic tree of hydrogenases and their specific proteases we suggest that a group 3 hydrogenase spread through HGT to the bacterial domain, probably together with a hydrogenase specific protease indicating that the proteolytic cleavage first evolved within group 3a/4 hydrogenases. We also propose that all 3d-type hydrogenases within bacteria evolved from this group 3 hydrogenase and therefore are the result of the same HGT event. Finally the novel observation of the so called HOXBOX may help in understanding the specificity seen among hydrogenase specific proteases and is an interesting target for further studies.

## Methods

### Bacterial strains and culture conditions

Cyanobacterial strains used in these experimental studies, *Nostoc *sp. strain PCC 7120 (also known as *Anabaena *sp. strain PCC 7120) [63], and *Nostoc punctiforme *ATCC 29133 (also known as *Nostoc *sp. strain PCC 73102) [64] were grown in BG11_o _medium (N_2_-fixing cultures) at 30°C under continuous light (40 μmol photons s^-1^m^-2^) and by sparging with air as previously described [65]. For non N_2_-fixing growth (cultures with no heterocysts) NH_4_Cl (2.5 mM) and MOPS (0.5 mM), adjusted to pH 7.8, were added to the medium. All cultures were mixed using a magnetic stirrer. *Escherichia coli *strains were grown in LB medium or on agar plates containing LB medium and antibiotics of interest at 37°C.

### RNA and DNA isolation

N_2_-fixing cell cultures were harvested in room temperature for DNA isolation as previously described [5] with the exception that 2 M instead of 3 M of NaAc was used. RNA was extracted from both N_2_-fixing and non N_2_-fixing cultures by centrifugation of the cells (4,500 × g for 10 in) in room temperature followed by resuspension in 1 ml TRIzol reagent (Sigma). The cells were then disrupted with 0.2 g of acid washed 0.6-mm-diameter glass beads by using a Fast-prep (Precellys^®^24) at a speed of 5.5 for 3 × 20 s, keeping the samples on ice in between runs. Phases were separated by centrifugation at 15,000 × g for 10 min at 4°C and the cleared solution was then transferred to new tubes and incubated at room temperature for 5 min. 0.2 ml of chloroform were added to the samples which were thereafter gently turned by hand for 15 s followed by a 2 min incubation at room temperature. The samples were then centrifugated at 15,000 × g for 15 min at 4°C and the upper obtained liquid phase was transferred to new tubes. The precipitation of the RNA was performed by adding 0.25 ml isopropanol and 0.25 ml of salt solution (0.8 M Sodium citrate and 1.2 M NaCl) followed by incubation at room temperature for 10 min. The RNA was then collected by centrifugation 15,000 × g for 10 min at 4°C and washed with 75% ethanol before treatment with DNase I (GE Healthcare) in 20 μl Dnase buffer (40 mM Tris-HCl, 6 mM MgCl_2_, pH 7.5) for 30 min at 37°C. A phenol: chloroform extraction was performed and the RNA was precipitated in 2.5 volume of ice-cold ethanol (99.5%) and 0.2 volume of cold LiCl (10 M). After precipitation at -20°C over night the samples were centrifuged at 20,000 × g, washed and resuspended in DEPC-treated distilled H_2_O.

### Identification of transcriptional start points (TSP)

TSP studies were performed using RNA from N_2_-fixing cultures and the "5'RACE System for Rapid Amplification of cDNA Ends" kit (Invitrogen) according to manual. Resulting bands were cloned into the pCR 2.1-TOPO vector (Invitrogen) and transformed into DH5α competent cells, all according to instructions from the manufacturer. The obtained vectors were purified by the "Genelute Plasmid Mini-prep Kit" (Sigma-Aldrich) followed by sequencing (Macrogen Inc).

In the case of *hoxW *in *Nostoc *PCC 7120, the primers used for the reactions were modified and designed according to the TAG-method [66] and only the first of the two nested PCRs described in the "5'RACE System for Rapid Amplification of cDNA Ends" kit manual was performed (Table 1).

**Table 1:** Primers used in this study.

Strain/Target	Oligonucleotide (name and sequence), 5'→3'		Primer pair	Product size (bp)
***Nostoc punctiforme *ATCC 29133**				
RT-Reaction				
*hupW- antisense*	HupW N R	TCA CAT CAT CGG GAA AGT CA		
Subsequent PCR				
*hupW-antisense*	HybD-RACE 1	TTC TGG CAA AGC TTC CAG TT	*L0373/L0372*	808/1361
*Npun_F0373-sense*	L0373	AAT TAT CTC CCT CGC GTT CC	*HybD-RACE 1*	808
*NpF0372-sense*	L0372	TTG CCG ATG AAA CAA ATG AA	*HybD-RACE 1*	1361
Northern blot, probe				
*hupW-antisense*	HupW N R	TCA CAT CAT CGG GAA AGT CA	*NB hupW N*	336
*hupW-sense*	NB hupW N L	TTG GTT GCG GAA ATC TCA AT	*HupW N R*	336
5'RACE				
*cDNA synthesis*	HybD-RACE 1	TTC TGG CAA AGC TTC CAG TT		
*1*^*st*^*PCR*	HybD-RACE 2	TGT TGG GCA ATG ATT ACA CCT		
*2*^*nd*^*PCR*	HybD-RACE 3	ATT GAG ATT TCC GCA ACC AA		
***Nostoc *sp. strain PCC 7120 – *hupW***				
RT-Reaction				
*hupW- antisense*	NB HupW- AR	TGC TGT AGG CGT AAT CAT CG		
Subsequenct PCR				
*hupW-antisense*	Alr1422-23 R	TTT GTA AGC GTT GAG CGA TG	*Alr1422-23 L*	490
*Alr1422-sense*	Alr1422-23 L	ACC GAA CTC CGC AGA AAC TA	*Alr1422-23 R*	490
5'RACE				
*cDNA synthesis*	ALR1423 RACE 1b	GTT CCG AAC CAG TGG AAC TC		
*1*^*st*^*PCR*	ALR1423 RACE 2	TTT GTA AGC GTT GAG CGA TG		
*2*^*nd*^*PCR*	ALR1423 RACE 3	GAG ATT TCC GCA ACC GAT AA		
***Nostoc sp. strain *PCC 7120 – alr1422**				
5'RACE				
*cDNA synthesis*	5-1422-1	CCTAAAGTCGGTGGAAAATCGGC		
*1*^*st*^*PCR*	5-1422-2	TTCTTCCGTGACAAATCGTG		
*2*^*nd*^*PCR*	5-1422-3	TTTTTGATGGACGGATGACA		
***Nostoc sp. strain *PCC 7120 – *hoxW***				
Northern blot, probe				
*hoxW-antisense*	NB HoxW A R	AAA GCG ATC GCC TAT TTC AA	*HoxW L*	316
*hoxW-sense*	HoxW L	AGG ACA ACG GAT AGC GAA TG	*NB HoxW A R*	316
5'RACE				
*cDNA synthesis*	5'RACE-1 HoxW/A	CAC AGC ACG ACG AAC AAG GCT CCA ACT TCA AAC CA		
*1*^*st*^*PCR-TAG*	5'RACE-TAG Hox/A	CAC AGC ACG ACG AAC AAG G	*5'RACE-polyG Hox/A*	
*1*^*st*^*PCR-PolyG*	5'RACE-polyG Hox/A	CAC AGC ACG ACG AAC AAG GGG GGG GGG GG	*5'RACE-TAG Hox/A*	

### Transcriptional studies

cDNA for transcriptional studies by RT-PCR were produced from RNA from N_2_-fixing and non N_2_-fixing cultures by using the RevertAid™ First Strand cDNA Synthesis Kit (Fermentas) containing RevertAid™ H Minus M-MuLV Reverse Transcriptase and RiboLock™ Ribonuclease Inhibitor according to the instructions. The following PCRs were done using TAQ polymeras (Fermentas) according to manufacturers instructions and visualized on a 1% agaros gel.

The probe used for Northern blot was produced by PCR amplification with appropriate primers (Table 1) and purified with the GFX, PCR, DNA and Gel Band Purification Kit (GE Healthcare). 7 μg of total RNA from N_2_-fixing and non N_2_-fixing cultures of *Nostoc *PCC 7120 and *Nostoc punctiforme *was separated by electrophoresis in denaturing agarose gels and blotted to Hybond-N+ (GE Healthcare) according to instruction using the, in the instruction described, modified Church and Gilbert buffer. Labelling of the probes was done using the Rediprime II Random prime labelling system (GE Healthcare) and removing of unincorporated ^32^P dCTP was thereafter performed by using Probe Quant G-50 microcolumns (GE Healthcare). The equal loading of the RNA was analyzed by the relative amount of *rnpB *transcripts. The positioning of the bands was visualized using a Pharos FX™ plus Molecular Imager (Bio-Rad) and analyzed with the accompanying software.

All primers used in this study were designed using the online primer program Primer3 [67,68] (Table 1).

### Protein and nucleotide sequence analysis and construction of phylogenetic tree

All strains and proteins, together with their GenBank accession number, used in this study are shown in Table 2[69-87]. Protein sequences used for the phylogenetic tree were retrieved from the NCBI database [88]. All alignments were performed in BioEdit version 7.0.4.1 [89] using ClustalW multiple alignment and the resulting alignment were corrected manually. For the construction of the unrooted phylogenetic tree the alignments were run through PAUP version 4.0 beta and MrBayes 3.1 software [90-92]. The maximum parsimony analysis (PAUP) was performed with heuristic algorithm and random addition of the sequences and bootstrap support values was calculated 1000 times. For the bayesian analysis MrBayes was executed for 1 000 000 generations with a sample frequency of 100 using the WAG model. A burn-in of 2500 trees was used and the support values indicate the proportion of the 7500 remaining trees. The online program ModelGenerator was used to determine the optimal model (WAG) [93,94]. For graphic outputs the resulting trees were then visualised by using Treeview [95,96].

**Table 2:** Microorganisms and genes used in this study.

Strain/Putative protease/Accession #	Abbreviation^a^	Proposed phylogenetic group	H_2_ase	Accession #	Ref.
*Acetomicrobium flavidum*/*hydD*/CAA56465	HydDAf	3d			
*Azoarcus sp. strain BH72/hupD*/YP_935294	HupDABH72	1			[78]
*Anabaena variabilis *ATCC 29413/*hoxW*/YP_325157	HoxWAv29413	3d			
*Anabaena variabilis *ATCC 29413/*hupW*/ABA23552	HupWAv29413	2			
*Desulfovibrio gigas*/hynC/*CAA11501*	HynCDg	1			[84]
*Desulfovibrio vulgaris *strain *Miyazaki F*/*hynC*/AAY90127	HynCDv	1	*hydB*	P21852	[69]
*Desulfovibrio vulgaris subsp. vulgaris *DP4/Dvul_1244/YP_966690	DvDP41	1			
*Desulfovibrio vulgaris subsp. vulgaris *DP4/Dvul_1247/YP_966693	DvDP42	1			
*Escherichia coli *K12/*hyaD*/NP_415494	HyaDEc	1			[83]
*Escherichia coli *K12/*hybD*/NP_417467	HybDEc	1	*hybC*	NP_417468.1	[83]
*Escherichia coli *K12/*hycI*/NP_417197	HycIEc	4			[83]
*Gloeothece *sp. strain PCC 6909/*hupW*/AAS72556.1	HupWG6909	2			[44]
*Lyngbya *sp. strain PCC 8106/*hoxW*/ZP_01622075	HoxWL8106	3d			
*Lyngbya *sp. strain PCC 8106/*hupW*/ZP_01619037	HupWL8106	2			
*Methanocaldococcus jannaschii *DSM 2661/*hycI*/NP_247615	HycIMj	4			[70]
*Methanocaldococcus jannaschii *DSM 2661/*frcD*/NP_246993	FrcDAMj	3a			[70]
*Methanocaldococcus jannaschii *DSM 2661/MJ0253/NP_247224	FrcDBMj	3a			[70]
*Methanococcus maripaludis *S2/*frcD*/NP_987939	FrcDMm	3a			[74]
*Methanococcus maripaludis *S2/*fruD*/NP_988503	FruDMm	3a			[74]
*Methanococcus maripaludis *S2/*HycI*/NP_988305	HycIMm	4			[74]
*Methanococcus maripaludis *S2/MMP1337/NP_988457	MmS2	3a			[74]
*Methanococcus voltae*/*frcD*/CAA43497	FrcDMv	3a			[73]
*Methanococcus voltae*/*fruD*/CAA43501	FruDMv	3a			[73]
*Methylococcus capsulatus *strain *Bath*/*hoxW*/YP_112652	HoxWMcB	3d			[86]
*Nitrosospira multiformis *ATCC 25196/*hoxW*/YP_412365	HoxWNm25196	3d			
*Nodularia spumigena *CCY9414/*hupW*/ZP_01628408	HupWNs9414	2			
*Nostoc punctiforme *ATCC 29133/*hupW*/YP_001864099	HupWN29133	2	*hupL*	YP_001864094	[81]
*Nostoc *sp. strain PCC 7120/*hoxW*/NP_484813	HoxWN7120	3d	*hoxH*	BAB72723.1	[63]
*Nostoc *sp. strain PCC 7120/*hupW*/NP_485466	HupWN7120	2	*hupL*	BAB72634.1	[63]
*Pyrococcus furiosus *DSM 3638/*hycI*/AAL80741	HycIPf	4			[79]
*Ralstonia eutropha *H16/*hoxM*/AAP85761	HoxMReH16	1			[85]
*Ralstonia eutropha *H16/*hoxW*/CAA63575	HoxWReH16	3d			[85]
*Ralstonia eutropha *H16/PHG070/AAP85823	ReH16	-			[15]
*Rhizobium leguminosarum bv. Viciae*/*hupD*/P27649	HupDRl	1			[75]
*Salmonella enterica subsp. enterica serovar Choleraesuis *str. SC-B67/*hyaD*/AAX65690	HyaDSe	1			[71]
*Salmonella enterica subsp. enterica serovar Choleraesuis *str. SC-B67/*hupD*/AAX65459	HupDSe	1			[71]
*Salmonella enterica subsp. enterica serovar Choleraesuis *str. SC-B67/*hybD*/AAX66993	HybDSe	1			[71]
*Salmonella enterica subsp. enterica serovar Choleraesuis *str. SC-B67/*hycI*/AAX66684.1	HycISe	4			[71]
*Shigella boydii *Sb227/*hyaD*/ABB66821	HyaDSb	1			[87]
*Shigella boydii *Sb227/*hybD*/ABB67388	HybDSb	1			[87]
*Shigella boydii *Sb227/*hycI*/ABB67327	HycISb	4			[87]
*Synechococcus *sp. strain PCC 7002/*hoxW*/AAN03570.1	HoxWS7002	3d			
					
*Synechocystis *sp. strain PCC 6803/*hoxW*/BAA17680.1	HoxWS6803	3d			[76,77]
*Thiocapsa roseopersicina/-/*AY214929	HoxWTr	3d			[72]
*Thiocapsa roseopersicina/hupD*/Q56362	HupDTr	1			[80]
*Thiocapsa roseopersicina/hydD-hynD*/AAN87047.1	HynDTr	-			[82]

^a^As used in phylogenetic tree (Figure 1).

Hydrogenases shown in the table do not represent the total number of hydrogenases in each organism.

Abbreviations; H_2_ase; hydrogenase, ref: reference.

Searches for homologues sequences of Npun_F0373 (*Nostoc punctiforme*), Alr1422 (*Nostoc *PCC 7120) and promoter regions were done by both using the NCBI and CyanoBase databases and their respective BLAST programs. Prediction of DNA secondary structure was done by using the online program MFold [97,98]. Transmembrane regions were predicted using the online program SOSUI [99-101].

For location studies of conserved residues on the surface of the proteases, alignments were performed for three of the protease groups revealed in the phylogenetic tree; group 5 – proteases of HoxW type (HoxW from *Nostoc *PCC 7120,*Anabaena variabilis *ATCC 29413,*Lyngbya *sp. strain PCC 8106, *Ralstonia eutropha *H16,*Thiocapsa roseopersicina, Synechococcus *sp. strain PCC 7002,*Synechocystis *sp. strain PCC 6803, *Mycobacterium vanbaalenii *PYR-1, and *Methylococcus capsulatus *strain *Bath*), group 2- cyanobacterial proteases of HupW type (HupW from *Nostoc *PCC 7120, *Nostoc punctiforme*, *Lyngbya *sp. strain PCC 8106, *Anabaena variabilis *ATCC 29413, *Nodularia spumigena *CCY 9414 and *Gloeothece *sp. strain PCC 6909) and group 1- proteases of HybD type (HupD/*Azoarcus *sp. BH72, HupD/*Bradyrhizobium japonicum*, HynC/*Desulfovibrio gigas*, HynC/*Desulfovibrio vulgaris *str. *Miyazaki F*, *Desulfovibrio vulgaris *subsp. *vulgaris *DP4, HyaD/HybD/*E. coli *K12, HoxM/*Ralstonia eutropha *H16, HupD/*Rhizobium leguminosarum bv. Viciae*, HyaD/HupD/HybD/*Salmonella enterica subsp*.*enterica serovar Choleraesuis *str. SC-B67, HyaA/HybD/*Shigella boydii *Sb227 and HupD/*Thiocapsa roseopersicina*). Conserved residues shared by 100%, 90%, and 80% of the sequences were then visualised on the surface of the 3D models on a representative from each group; the 3D models of HoxW and HupW from *Nostoc *PCC 7120 and on the crystallized structure of HybD from *E. coli *(protein data bank accession number 1CFZ.pdb).

### 3D modelling and protein docking

3D models of proteases were constructed by using the online program SWISS-MODEL [102] and with HybD from E. coli as a template (1CFZ.pdb). The same method were also used for the 3D models of the large subunits of the hydrogenases, using HydB from Desulufovibrio vulgaris Miyazaki F as template (protein data bank accession number 1UBJ:L). The results were visualised in the program Swiss-PDB-viewer [103,104].

Protein-protein docking simulations were done by using the docking program BiGGER V2 [105]. The following constraints were set; Gln16 and His93 in the protease had to be at a minimum distance of 8 Å from the Cys61 and Cys546 in the hydrogenase large subunit (amino acid numbers refers to HybD and HybC in E. coli). The docking experiments were then run as soft docking with an angular step of 15° and a minimum contact of 300. The residues used for constraints were chosen since they are suggested to bind to the nickel in the active site of the large subunit of the hydrogenase [17,62,106]. The docking simulations were done for the following combinations; HybC model – HybD (1CFZ) (E. coli), HydB (1UBJ:L) – HynC model (Desulfovibrio vulgaris str. Miyazaki F) and HoxH model – HoxW model (Nostoc PCC 7120). The best solutions were selected according to the global score from BiGGER V2 and with regard to the possibility of nickel binding.

## Authors' contributions

ED performed most experimental work; Most of the transcriptional studies of *hupW *and *hoxW*, all studies done in silico including phylogenetic studies and specificity studies and analysis of the data. She is the primary author of the final manuscript. MH identified the TSPs of alr1422/*hupW *in *Nostoc *PCC 7120. KS supervised the experimental work and was also involved in parts of the writing of the manuscript. PL conceived and coordinated the project and the manuscript. All authors have read and approved the manuscript.

## Supplementary Material

Additional file 1 **Supplementary extended tree. **This PDF-file contains an extended phylogenetic tree containing more hydrogenase specific proteases from both bacterial and archaean strains including putative type 3 b proteases. The proposed subgroups for each protease are marked in the figure; 1 (red), 2 (orange), 3a (blue), 3d (purple), 4 (green). When protease subgroup is unknown the group number of proposed cleavage substrate (hydrogenase) is written in brackets. It is based on the protease's placement within the phylogenetic tree, the number of hydrogenases within each strain and the possibility for co-transcription with a hydrogenase. X: The point in the phylogenetic tree when horizontal gene transfer might have occurred. Y/Z: Suggested positions of root. Archaean strains: red text. Bacterial strains: black text. For abbreviations used see Additional file 2. The tree were constructed using the MrBayes software which was executed for 1 500 000 generations with a sample frequency of 100 using the WAG model. A burn-in of 3750 (25%) trees was used. For graphic outputs the resulting trees were visualised by using Treeview. Click here for file

Additional file 2 **Table organisms.** This excel-file contains a table of all hydrogenase specific proteases used in the extended phylogenetic tree (Additional file 1) including strain, organism, locus_tag, abbreviation, accession number, and proposed phylogenetic group. This file also contains the number of hydrogenases in each strain including accession number. Proposed cleavage substrate (hydrogenase large subunit) for each protease is marked with **grey background/bold text **and is based on each protease position in phylogenetic tree, the number of hydrogenases within each strain and location within genome (i.e. possibility for co-transcription with hydrogenase gene). B; unknown phylogenetic group. Click here for file

Additional file 3 **Alignment NpunF0373homolgoues.** This word document file shows an alignment of NpunF0373 and homologues found in other organisms, all cyanobacterial strains, including locus_tag and accession number. Click here for file

Additional file 4 **Supplementary figure NpunF0373homologoues.** This word document file show the presence/absence of homologous to the gene Npun_F0373 of *Nostoc punctiforme *in selected cyanobacterial strains together with their, when present, locus_tag and GenBank accession number. *hupL*, *hupW*, *hoxH*, *hoxW *and different metabolic functions; the ability to produce heterocyst and filaments and the capacity for nitrogen-fixation, are also indicated. (+); present, (-); absent, (?); presence/absence unknown. Click here for file
