# Supplementary figures and images for: Diversity and transcription of proteases involved in the maturation of hydrogenases in Nostoc punctiforme ATCC 29133 and Nostoc sp. strain PCC 7120
Source: BMC Microbiol. 2009 Mar 11;9:53. doi: 10.1186/1471-2180-9-53 (PMC2670836; doi:10.1186/1471-2180-9-53)

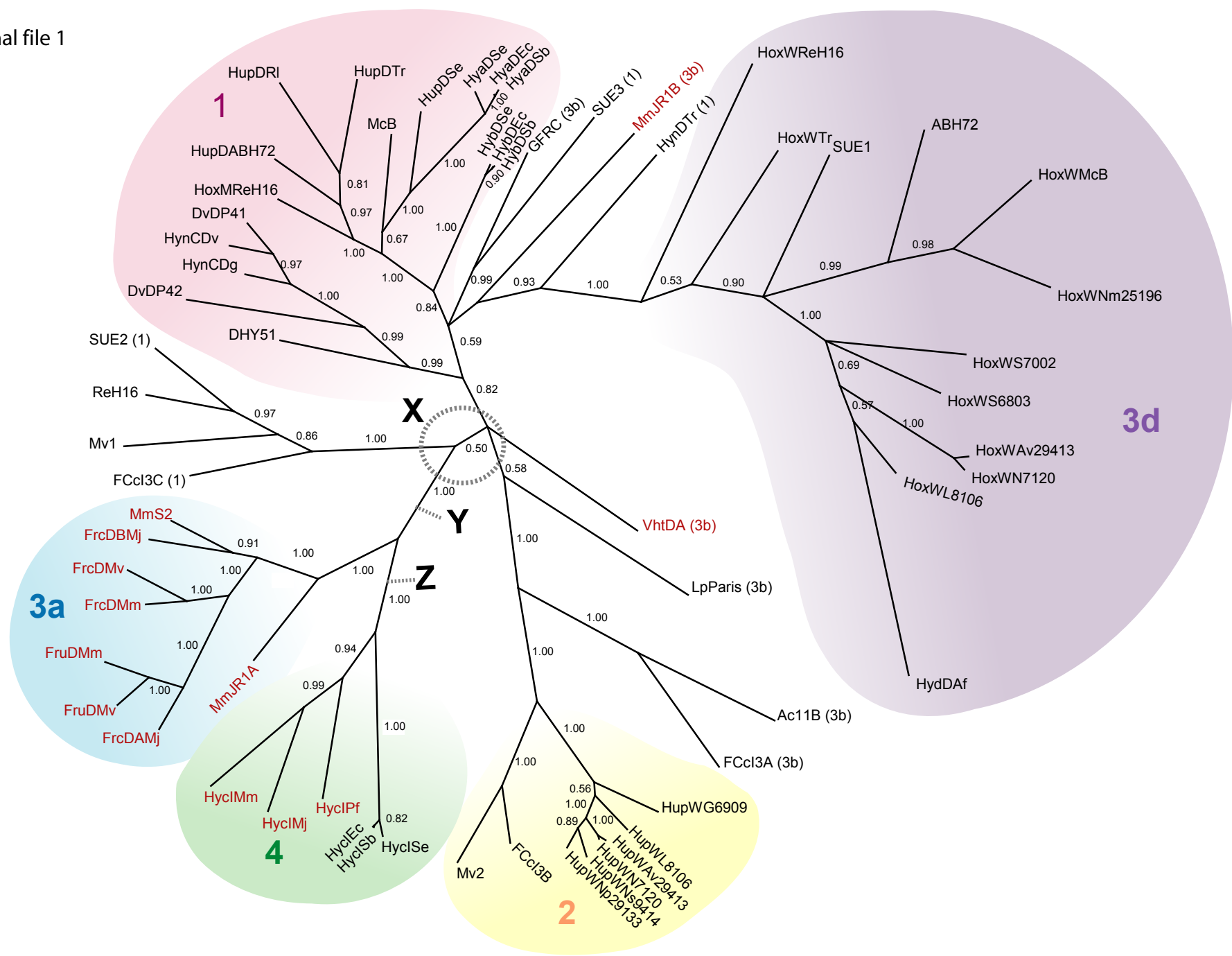

Supplement: Additional file 1 — Supplementary extended tree. This PDF-file contains an extended phylogenetic tree containing more hydrogenase specific proteases from both bacterial and archaean strains including putative type 3 b proteases. The proposed subgroups for each protease are marked in the figure; 1 (red), 2 (orange), 3a (blue), 3d (purple), 4 (green). When protease subgroup is unknown the group number of proposed cleavage substrate (hydrogenase) is written in brackets. It is based on the protease's placement within the phylogenetic tree, the number of hydrogenases within each strain and the possibility for co-transcription with a hydrogenase. X: The point in the phylogenetic tree when horizontal gene transfer might have occurred. Y/Z: Suggested positions of root. Archaean strains: red text. Bacterial strains: black text. For abbreviations used see Additional file 2. The tree were constructed using the MrBayes software which was executed for 1 500 000 generations with a sample frequency of 100 using the WAG model. A burn-in of 3750 (25%) trees was used. For graphic outputs the resulting trees were visualised by using Treeview. [file 1471-2180-9-53-S1.pdf]
