# Supplementary material for: Diversity and transcription of proteases involved in the maturation of hydrogenases in Nostoc punctiforme ATCC 29133 and Nostoc sp. strain PCC 7120
Source: BMC Microbiol. 2009 Mar 11;9:53. doi: 10.1186/1471-2180-9-53 (PMC2670836; doi:10.1186/1471-2180-9-53)
Supplement: Additional file 3 — Alignment NpunF0373homolgoues. This word document file shows an alignment of NpunF0373 and homologues found in other organisms, all cyanobacterial strains, including locus_tag and accession number. [file 1471-2180-9-53-S3.doc]

| **Gene** | **Strain** | **Alignment** |
| --- | --- | --- |
|  |  | ....|....| ....|....| ....|....| ....|....| ....|....| ....|....| ....|....|  10 20 30 40 50 60 70 |
| All1395 | *Nostoc* PCC 7120 | MSDLELHKYL PKLPETALQE FTEWCVVEQS RAAGIEFIPD KTKLEKLIPN EYIWQIIDQF MKAKPDPIKA |
| NpF0373 | *Nostoc* PCC 29133 | VSDLALHNYL PRVPDAALQE YIEWCVLEQA QAAECNFTPD RSKLDNLLPE DYIPKLVEQF MKVKPDPIKA |
| - | *Nostoc* PCC 7422 | VSDLALHNYL PRVPDAALQE F …*Not sequenced.* |
| Ava3950 | *Anabaena variabilis* ATCC 29413 | MSDLELHKYL PKLPETALQE FTEWCVVEQS RAAGIEFIPD KTKLEKLIPN EYIWQIIDQF MKSKPDPIKA |
| N9414_14940 | *Nodularia spumigena* CCY9414 |  |
|  |  | *** ** ** * * **** **** ** ** * ** ** * * ** ** ** ******* |
|  |  | ....|....| ....|....| ....|....| ....|....| ....|....| ....|....| ....|....|  80 90 100 110 120 130 140 |
| All1395 | *Nostoc* PCC 7120 | GLVSAIAGQE ADSHGLVGSA IMVDFISLYV KYLIPENGNT PEEAKQLILE AAIQQYEKLS ELADKYNVKF |
| NpF0373 | *Nostoc* PCC 29133 | GLVAAIAGKE ADKHALSGLA IAADFVSLYV KYLIPKEGST KEQAEEILTQ ASQHQYEKLT EVAKKHGVEF |
| - | *Nostoc* PCC 7422 | *Not sequenced* |
| Ava3950 | *Anabaena variabilis* ATCC 29413 | GLVSAIAGQE ADSHGLVGSA IMVDFISLYV KYLIPENGNT PEEAKQLILE AAIQQYEKLS ELADKYNVQF |
| N9414_14940 | *Nodularia spumigena* CCY9414 | GKE ADKHGLSGLA VVADFIALYV KYLIPKDGNT PQQAEMILTD ASQKQCEKFT EIAKKYDVKF |
|  |  | *** **** * ** * * * * ** *** ***** * * * * * ** * * * * * |

**Additional file 3**

Conserved residues are marked by asterisks, conserved substitutions by double points and semi-conserved substitutions by points.

;Transmembrane region
