# Supplementary material for: Diversity and transcription of proteases involved in the maturation of hydrogenases in Nostoc punctiforme ATCC 29133 and Nostoc sp. strain PCC 7120
Source: BMC Microbiol. 2009 Mar 11;9:53. doi: 10.1186/1471-2180-9-53 (PMC2670836; doi:10.1186/1471-2180-9-53)
Supplement: Additional file 4 — Supplementary figure NpunF0373homologoues. This word document file show the presence/absence of homologous to the gene Npun_F0373 of Nostoc punctiforme in selected cyanobacterial strains together with their, when present, locus_tag and GenBank accession number. hupL, hupW, hoxH, hoxW and different metabolic functions; the ability to produce heterocyst and filaments and the capacity for nitrogen-fixation, are also indicated. (+); present, (-); absent, (?); presence/absence unknown. [file 1471-2180-9-53-S4.doc]

**Additional file 4**

| **Strain** | **Locus_tag** | **Accession #** | **Heterocyst** | **Filament** | **N_2_**  **fixing** | ***hupL*** | ***hupW*** | ***hoxH*** | ***hoxW*** |
| --- | --- | --- | --- | --- | --- | --- | --- | --- | --- |
| *Nostoc punctiforme* ATCC 29133 | Npun_F0373 | YP_00186409098 | **+** | **+** | **+** | **+** | **+** | **-** | **-** |
| *Anabaena variabilis* ATCC 29413 | Ava3950 | YP_324450 | **+** | **+** | **+** | **+** | **+** | **+** | **+** |
| *Nostoc* sp. strain PCC 7120 | All1395 | NP_485438 | **+** | **+** | **+** | **+** | **+** | **+** | **+** |
| *Nodularia spumigena* CCY9414 | N9414_14940 | ZP_01628407 | **+** | **+** | **+** | **+** | **+** | **+** | **+** |
| *Nostoc* sp. strain PCC 7422 | - | AB237640 | **+** | **+** | **+** | **+** | **?** | **+** | **?** |
| *Lyngbya* sp. strain PCC 8106 |  | **-** | **-** | **+** | **+** | **+** | **+** | **+** | **+** |
| *Gloeothece* sp. strain PCC 6909 |  | **-** | **-** | **-** | **+** | **+** | **+** | **?** | **?** |
| *Synechocystis* sp. strain PCC 6803 |  | **-** | **-** | **-** | **-** | **-** | **-** | **+** | **+** |
